# Temporal and Spatial Characteristics of Pacing Strategy in Elite Women’s 400 Meters Hurdles Athletes

**DOI:** 10.3390/ijerph19063432

**Published:** 2022-03-14

**Authors:** Janusz Iskra, Krzysztof Przednowek, Jarosław Domaradzki, Milan Coh, Paweł Gwiazdoń, Krzysztof Mackala

**Affiliations:** 1 Faculty of Physical Education and Physiotherapy, Opole University of Technology, 45-758 Opole, Poland; j.iskra@po.edu.pl; 2 Institute of Physical Culture Sciences, Medical College of Rzeszów University, 35-959 Rzeszów, Poland; krzprz@ur.edu.pl; 3 Faculty of Physical Education and Sport, Wroclaw University of Health and Sport Science, 51-612 Wrocław, Poland; jaroslaw.domaradzki@awf.wroc.pl; 4 Faculty of Sport, University of Ljubljana, 1000 Ljubljana, Slovenia; milan.coh@fsp.uni-lj.si; 5 Faculty of Physical Education, Academy of Physical Education in Katowice (Doctoral Studies), 40-065 Katowice, Poland; pgwiazdon@wp.pl

**Keywords:** 400 mH run, pacing strategy, asymmetry, temporal and spatial variables, multidimensional statistics

## Abstract

The main objective of the study was to assess the pacing strategy of running 400 m hurdles of the world-level female athletes over the past 40 years based on the functional asymmetry -temporal and spatial characteristics. The data were collected from 1983 to 2019 using the review of scientific literature. Over the 35 years of the study, 37 top-level competitions with 283 finalists-competitors were included. The analysis of the 400 m hurdle covered mainly spatial and temporal factors of the run, related to those technical skills, the level of motor skills, and somatic structure. In addition to the basic statistics, the ANOVA analysis of variance, regression analysis, Pearson correlation, the principal component analysis (PCA), and Kaiser’s criterion was used for the multivariate analysis. The final result in the 400 mH run is determined not by the simple sum of the individual temporal and/or spatial characteristics of the run (the number of steps, the type of attacking leg, but their interaction in the area of functional asymmetry. The decisive factor in the 400 mH run strategy is the second curve, where the emphasis is on the optimal setting of the stride pattern in the context of minimizing the loss of running speed. Additionally, the application of multidimensional statistical methods is a valuable tool that allows to significantly deepen the interpretation of the obtained results, and thus optimize a strategy for a 400 mH run.

## 1. Introduction

The analysis of asymmetry in the area of biomechanics is useful in assessing the effectiveness and optimizing the performance of a specific motor structure, thus reducing the risk of injury [1,2,3]. This mainly applies to the so-called functional asymmetry between the limbs, e.g., differences in the performance of one limb’s movement structure about the other [4,5]. Therefore, asymmetry is part of contemporary sports training, implemented in running competitions, and it concerns mainly the time-space characteristics of a single running step, repeated many times [6,7,8], e.g., in a 400 mH run. Asymmetry (morphological, functional, gait, and fundamental movement patterns) is also related to the health of well-trained runners. Linkage with injuries is well-documented and asymmetry is one of the main intrinsic factors of acute and recurrence injuries [3,8,9,10].

The 400 m hurdles race is one of the most difficult athletic competition events [11]. The final result is determined both by the level of motor skills such as speed, anaerobic endurance strength [12,13,14,15], and the technical ability to overcome the hurdles [16,17]. A high level of motor skills and technical skills directly affects a specific skill called “hurdle rhythm”. This rhythm is defined as running with the minimum loss of speed, regardless of the fatigue and pattern of clearing the subsequent hurdles [18,19,20]. In most cases, it determines success in the hurdle race. Additionally, this rhythm is related to the so-called 400 mH pacing strategy. Attempts to explain this strategy as an effort optimization began in the 1960s, mainly on the analysis of the most important international competitions [20,21,22]. It includes the application of a certain number of steps between hurdles (15–17) and time variables to cover the distance between these hurdles [23,24].

The 400 m hurdles pacing strategy should include the symmetry aspects, regardless of the sport level at which it is implemented. In this case, symmetry is taking into account two interdependent factors. One concerns the temporal aspects-time divisions (splits) in particular sections of the race: the first turn, the first straight line, the second turn, the second straight line. The second aspect refers to the spatial elements defined as the number of steps between successive hurdles (from 1 to 10 hurdle). These primary factors can be extended to additional factors including the increase in the number of steps (e.g., from 13 to 14), the multiple of these changes in a single run, the ratio of the odd (13,15) to even (14) steps, and the use of the attacking leg-left or right [23,25,26].

Detailed and in-depth analysis of the sports movement observations and the resulting conclusions, requires the application of more and more advanced statistical analyzes. In particular, it concerns the analysis of the time and spatial series of sports movement to extract the features of the movement, that will optimize the result of the sport. In our case, the final time in the 400 mH race. The multidimensional relationships between measured variables determining the final result require the usage of multivariate statistical analyses. The article proposes a multidirectional statistical analysis (e.g., the principal component analysis (PCA), ANOVA, regression analysis, correlation) of hurdles observation, which relates to the analysis of changes in spatial attributes (e.g., number of steps, distance traveled), and non-spatial attributes (e.g., speed and time of crossing each hurdle). This happens in terms of functional symmetry. Therefore, the main aim of this study was to investigate the pacing strategy in Women’s 400 m hurdles, studying functional symmetry. Specifically, we aimed to: (1) study the basic temporal and spatial characteristics of pacing strategy in female 400 m hurdles athletes, (2) overview the relationship between temporal and spatial characteristics, (3) investigate variables of the greatest importance for the final result, (4) identify main components of strategy by reducing the dimensionality of data set, (5) assess differences between female groups varying sports level.

## 2. Materials and Methods

### 2.1. Material

Two hundred and eighty-three female 400 mH runners belonging to 23 national teams around the world were analyzed (age: 26.05 ± 3.8 years; body height: 172.57 ± 5.09 cm; body mass: 59.74 ± 4.33.2 kg). One of the statistical analyzes included the division of runners into three groups. Differences between the groups were confirmed by comparing mean personal best times in the official 400 m H race: Group A, 53.38 ± 0.42; Group B, 54.51 ± 0.29; and Group C, 55.83 ± 0.67. The study was approved by the Human Ethics Committee of the Opole University of Technology, Opole, Poland (U-KO-212/2021/PO and 25 March 2021).

### 2.2. Methods

Data acquisition was performed through computer searches of scientific literature for the years 1978–2019 using several primary sources of information: Medline (PubMed), Web of Science, Evidence Database (PEDro), EMBASE, Scopus, and Google Scholar. The search used the following keywords: “400 mH race”. Moreover, this descriptive review was performed by the Preferred Reporting Items for the review statement (http://www.prisma-statement.org, accessed on 15 September 2021). The literature review made it possible to obtain titles and summaries of all publications included in the keyword. This allowed for the isolation of publications for inclusion so that the allocation criteria, the women’s 400 mH run in the articles, were met. In this way, the full texts of the manuscripts for the current review were obtained. Only studies focused on 400 mH strategies or pacing strategies in championship competitions are included in the manuscripts. Articles that analyzed data obtained from local competitions or national championships were excluded. The final analysis included 283 individual runs at championship competitions (Table 1). All the races concerned were event finals, the competition was held on synthetic tracks, and the time was officially measured with an accuracy of 0.01 s. The data (time and space) were obtained from materials published over the last 40 years.

Variables from three areas were presented in a detailed statistical analysis:

1. Variables characterizing the body structure (BH, BW, BMI) and the level of motor skills (PB 400 m) and technical skills (Technical Index = PB 400 H-PB 400). Additionally, the age of female runners was taken into consideration.
2. Time variables (“split times”)—times of individual sections between hurdles (*t*_1–2_, *t*_2–3_, … *t*_9–10_) and times of three characteristic parts of the run (*t*_1–4_, *t*_4–7_, *t*_7–10_). Additionally, the run-up time to the first hurdle (*t*_0–1_) and the finish section time (*t*_10_) were taken into account.
3. Spatial variables (“stride pattern”, i.e., the number of steps between successive hurdles)—as in the case of time parameters, successive sections (*n*_1–2_, *n*_2–3_, … *n*_9–10_) and selected parts were taken under consideration (*n*_1–4_, *n*_4–7_, *n*_7–10_).

### 2.3. Statistical Analysis

The normality of distributions was assessed using the Shapiro–Wilk test. In the presentation of results, basic statistical measures were used, i.e., arithmetic means, standard deviation, minimum and maximum variables, kurtosis, and skewness. The relationships between temporal/spatial parameters and final results were analyzed using the Pearson correlation.

To assess the influence of the tested parameters on the result of the 400 m hurdles, a stepwise forward regression was used. The procedure begins with an equation that contains only a free expression. The first variable in the equation is the one that has the highest correlation with the *Y* variable. The variable remains in the equation if the coefficient of regression of the variable differs significantly from zero. The next variable introduced into the equation is the one that has the highest correlation with *Y*. (*Y* has been adjusted for the effect of the first variable). If the regression coefficient is significant, adding the next variable is implemented in the same way.

In studies carried out to reduce the number of variables characterizing the results in 400 m hurdles, the principal component analysis (PCA) was used. The PCA method involving matrix operations is used for multidimensional data exploration, projection, and visualization. PCA analysis results in new principal components which are linear combinations of vectors subjected to analysis regarding maximization of variance description. Reduction of data dimensionality is carried out by studying the acquired eigenvalues of principal components to describe the percentage of described data variance. In this study, Kaiser’s criterion is used, which eliminates principal components of eigenvalues of less than 1 from further analysis [4].

The ANOVA test was used to assess the differences of selected variables between groups with different sport levels (high, medium, low). If statistically significant differences were identified, then detailed posthoc comparisons were calculated. In all analyses, statistical significance was set on *p* = 0.05 (calculated *p* < 0.05 was recognized as statistically significant). All analyses were carried out with the use of the R programming language with additional packages (R Core Team, 2018).

## 3. Results

Studied female athletes were heterogeneous terms of age. It suggested mean of anthropometric measurements suggested a normal range of body height to body mass proportions. However, the minimum–maximum range of BMI indicated underweighted participants including in studies, whereas wide ranges of min-max of sport results suggested differences in sports level of participants. It allowed separating several groups of athletes on different sports levels for her part of the analysis. The most variation of results presented time index (42.31% of variability) compared with 400 mH times and PB 400 m (Table 2).

Temporal and spatial characteristics of the pacing strategy are presented in Table 3, Table 4 and Table 5. Changes in basic time variables (*t*_1,2,3…_) indicate a systematic reduction in running speed, assessed by the time of covering nine 35 m sections (Table 3). Each of the above races is important for the final results. The most significant temporal parts of the run are the so-called “entrance in the bend” and “leaving the bend”. Their significance is confirmed by bivariate correlations (*t*_5–6_, *r* = 0.73 and *t*_8–9_, *r* = 0.75; Table 3), whereas the most significant spatial characteristics are *nt*_5–6_ and *nt*_6–7_ (*r* = 0.36 and *r* = 0.37, respectively) (Table 4).

The changes in the stride pattern (stride number) in this group were found in the second turn (*n*_4–7_). Running speed losses were significant in its final part *r*(*t*_7–10_–*t*_1–4_) = 0.92; *p* < 0.001). Hurdlers who performed well in terms of the fast initial section of the race (=“speed” hurdlers) applied a 15-step rhythm at the start of the race, and then changed to 16 strides later in the distance. The greatest changes in the stride pattern were found in the last section of the race *n*_7–10_–*n*_1–4_, where runners applied mainly 17 steps between hurdles. The use of the “speed” strategy lengthened the split time in the section of the final race (from the 7th hurdle) (Table 5).

The next step in the analysis was to review the Pearson product correlation coefficients with the moment examining the linear relationship between all temporal and spatial parameters. The results presented in Table 6 showed the strongest and positive relationships between *t*_1–4_ and *t*_4–7_ among time variables and *n*_1–7_ and *n*_4–7_ among spatial variables. Moderately positive associations were also observed between *t*_4–7_ and *n*_4–7_ as well as *t*_7–10_ and *n*_7–10_ (*r* = 0.39 and *r* = 0.40, respectively).

Stepwise forward regression analysis was then conducted to identify the variable the most important for the final results of the 400 m hurdles. Among the analyzed parameters, 7 variables reached a statistically significant connection with the result in the 400 m hurdle race (Table 7). The greatest amount of information is derived from variables (according to standardized *r* coefficients): *t*_7–10_, *t*_4–7_, and *t*_10–F_. So, three temporal parameters are key for best results in Women’s 400 m hurdles. Times for the individual parts of the run, with an emphasis on the middle (*t*_4–7_) and the final (*t*_7–10_) parts. The regression analysis also shows the importance of the running technique index (TI) and the number of steps in its central part (*n*_4–7_). The chosen regression model explains approximately 96% of the problem (Table 7).

The PCA analysis allowed for the identification of 6 factors explaining together 76.82% of the variance. The first three factors characterize the time parameters, successively*-*the third (23.36%), the first (17.79%), and the second (11.00%) part of the distance. In terms of factor no. 1, the number of steps in the last part of the run was included (−0.70) as well as all differences in the increase in the number of steps (Table 8). In the case of factor no. 2, the share of parameter *n*4–7 is noteworthy. This proves the dependence of the first two parts of the run.

The analysis of variation (ANOVA) indicates further determinants of the running strategy. The data show that body height does not influence the results (Table 9). Time differences between groups with different sports levels are significant (*p* ≤ 0.001), while the “stride pattern” differences are connected mainly with the middle part of the run (*n*_4–7_). The critical part of the run is the section between the fourth and seventh hurdle, while the changes in the “stride pattern” in the final part of the distance (*n*_7–10_–*n*_4–7_) are irrelevant.

## 4. Discussion

This study aimed to investigate the pacing strategy in Women’s 400 m hurdles, studying functional symmetry in different special and temporal conditions. This mainly applies to the number of steps taken between hurdles in individual phases of running*-*straight and curved, and the leg that attacks the hurdle: left or right. In turn, the number of steps taken depends on the optimal functionality of the lower limb—the limb that attacks the hurdle: left or right. Detailed analysis showed some important trends. Changes in the running speed of modern hurdles over a distance of 400 m are characterized by a reduction in speed after the first hurdle. The “rhythm as several steps” in the first part of the run is mainly 15 steps between hurdles, up to 5–6 hurdles, which is already a standard. Additionally, the decisive factor in the 400 mH run strategy is the second curve, with an emphasis on the optimal linkage of the stride pattern in the context of minimizing the loss of running speed by adding one step and attacking the hurdle from the opposite leg.

The analysis of changes in running speed (=time to cover successive sections between obstacles) and differences in the number of steps (“step pattern”) prove the effectiveness of the 400 m hurdles strategy [27]. Therefore, the course of changes in running speed over a distance of 400 m hurdles should be qualified as a preliminary assessment of the running strategy implemented by the competitor [26,28,29,30]. Some studies indicate a gradual reduction in running speed after defeating the third or fourth hurdle, usually attacked with the “efficient leg” and with a 15-step rhythm between the second and fourth hurdle. Analyzes of data from recent years have shown, however, that modern female runners start to run very fast, and after the second hurdle the running speed begins to decrease (Table 3). As suggested by some authors [31,32], the differences in the speeds developed at the beginning of the run determine the appropriate running strategy, characterized by the speed level in subsequent sections of the distance (second bend, second straight). Most often, however, these changes concern the end of the first straight line (*t*_3–4_), and not, as in our research, the beginning (*t*_2–3_).

The so-called “stride rhythm” plays a significant role in this analysis. It is an indispensable element in creating a running strategy. For most authors, the analysis of the first part of the distance is characterized by a constant number of steps: 15 or 16 [28,33,34]. Our analyzes confirmed these data—the best female runners in the world cover the first part of the distance in the “rhythm” of 15 steps (Table 5). This, however, causes the speed reduction later in the run forces the competitors to change the number of steps between the hurdles from the sixth to the last hurdle (Table 4). This is usually manifested by adding one step on the second bend (16 steps) and the attack of 2–3 hurdles the opposite leg to the more efficient one [34,35,36]. On the second straight we have the next step, e.g., 17, and keep this rhythm until the finish. This action clearly shows the importance of functional symmetry: the number of steps, the leg, the distance running phase.

However, the use of various statistical methods allowed for the clarification and better understanding of what depends on obtaining the optimal strategy in women’s 400 m run, with particular emphasis on the influence of asymmetry. This is significant not only from the differences in the results obtained but the number of steps used between the hurdles and the number of changes in the attacking leg from left to right and vice versa.

So far, the most information regarding the choice of a running strategy at 400 mH has been provided by the analysis of the correlation of selected parameters with the final result [37]. Some authors [12,14,30,38,39] indicate that in addition to the technique of overcoming the hurdle, the level of anaerobic endurance, assessed with the result of running at 400 m flat, is significantly related to running time at 400 mH (Table 2). However, the analyses carried out on a larger number of publications indicate a decrease in this relation (Table 6), as well as the lack of significant correlation between the final result and the Technical Index (*r* = 0.10, NS). These data confirm the new approach to women’s 400 mH running, emphasizing the importance of curve running and the relevance of this element in teaching and training [24,25,38]. This justification is fully illustrated by the correlation analysis in Table 4 and Table 5. Faster running in the initial part of the track, causes the speed of the runner to slow down in the final section (*rt*_0–1_/*t*_10–*F*_ = −0.30). The connection of the running time with the number of steps performed is of particular importance in the second part of the distance (*rt*_4–7_/*n*_4–7_ = 0.39, *rt*_7–10_/*n*_7–10_ = 0.40) (Table 6). The correlation analysis also shows the significance of the number of steps performed in successive hurdle units. The beginning of the second turn (*t*_5–6_) and final (*t*_8–9_) part is decisive as it concerns the final result, and the sequence of steps (*n*_5–7_) on the second curve shows the greatest relationship with the sports proficiency. It is particularly important to optimally link changes in the “rhythm of steps” in the second part of the run with a gradual decrease in speed (Table 6). This problem of running strategy is emphasized by coaches and scientists [16,19,40]. Additionally, the regression analysis emphasizes the decisive importance of time parameters and (indirectly) spatial parameters (Table 7). The results of the analysis are consistent with the concepts of trainers who treat the “rhythm” of steps as a preliminary value (although necessary) in building a running strategy [16,39].

After identifying single variables, the most important for final results reducing the dimensionality of the original data set was the next step of work. Reducing the number of variables into a smaller set that still contains most of the information in the larger set has very practical applications. Statistically significant loading in each factor informs about the individual contribution of the variable in the constructed principal component. The use of factor analysis allows a search for common elements for the problem to be solved. This method is used in many sports [41], including hurdles [42]. Taking into account the basic parameters (including the level of motor and technical preparation), time and space, six factors were distinguished (Table 8). The most important factor is the parameter of the final part of the run. Important information for the development of a 400 m H run strategy has a factor of 3, suggesting the unique nature of the run between the fourth and seventh hurdles. Results of principal component analysis (PCA) are shown in Table 8.

Female runners presented in our analysis were divided into various sports proficiency groups. Therefore, all individuals were divided into three groups: the highest sports level (A), medium level (B), and runners with the lowest results (C). This division allowed for another/different assessment of the applied strategy of running Women’s 400 mH. One-way ANOVA was employed to achieve this objective (Table 9). The use of analysis of variation (ANOVA) in the evaluation of the running strategy is not often used in running/hurdling studies. Most often it concerns experimental studies assessing female runners divided according to various criteria [43]. The analysis of the differences of three groups of female runners with different levels of advancement proves that the most important parameters are time and space parameters of the second and third parts of the distance (Table 6). When body height was added to these two parameters, ANOVA showed that body height does not influence the results (Table 9). However, according to most studies, body height is the initial (often fundamental) element in developing a running strategy [14,19,20,27,44]. The changes that take place between the finish section and the mid-distance run determine the sports level of the hurdlers.

Summarized, the spatial parameters (=stride pattern) are the basic introduction to the realization of the run’s time structure (=split times), which determines the strategies of Women’s 400 mH run. Changes in the running speed of modern female 400 m hurdlers are characterized by a reduction in speed after the first hurdle. It is strongly related to the use of the so-called “speed” strategy, which lengthened the split time in the section of the final race (from the 7th–10th hurdle). The “rhythm as several steps” in the first part of the run is set up on 15 steps between hurdles, up to 5–6 hurdles, which can be qualified as a standard. The decisive factor in the 400 mH run strategy is the second turn, with an emphasis on the optimal linkage of the stride pattern in the context of minimizing the loss of running speed by adding one step and attacking the stride from the opposite leg.

Taking into account the results of the PCA analysis, most parameters assessing running strategies include the first factor of the “first part of the distance”, and the most specific factor concerns the middle part of the run. The difference in time and space parameters between the last and the middle part of the run is the basic element that differentiates female hurdlers with a different sports level.

From a practical point of view, to determine the optimal strategy for 400 mH (both: women and men), for each runner, the coach need to use additional tools*-*advanced statistical analysis. The final result is determined not by a simple sum of individual temporal and/or spatial characteristics, but by their mutual relationships at the discretion of functional asymmetry. Therefore, the multivariate methods are valuable tools that allow you to significantly deepen the interpretation of the obtained results.

## 5. Conclusions

The final result in the 400 mH run is determined not by the simple sum of the individual temporal and/or spatial characteristics of the run (the number of steps, the type of attacking leg, but their interaction in the area of functional asymmetry). The difference in the interaction of temporal and spatial parameters between the last and the middle part of the run is a significant factor differentiating the level of a Woman’s 400 mH run. The decisive factor in the 400 mH run strategy is the second curve, where the emphasis is on the optimal setting of the stride pattern in the context of minimizing the loss of running speed. The most common expression of this is adding one step and attacking the hurdle with the opposite leg (asymmetrical to more proficient).

From a practical standpoint, the application of multidimensional statistical methods are valuable tools that allow coaches, athletes, and professionals to significantly deepen the interpretation of the obtained results, and thus prepare a strategy for a 400 mH run that is optimal for each competitor.

## Figures and Tables

**Table 1:** Types of competitions, years of the competition, and the number of competitors participating in the final of the 400 mH race.

Competitions	Years	Athletes
Olympic Games (9)	1984, 1988, 1992, 1996, 2000, 2004, 2008, 2012, 2016	70
World Championships (15)	1983, 1987, 1991, 1993, 1995, 1997, 1999, 2001, 2003, 2005, 2007, 2009, 2011, 2015, 2017	112
European Championships (11)	1978, 1982, 1986, 1990, 1994, 1998, 2002, 2006, 2019, 2012, 2014, 2016	85
Friendship Games (1)	1984	8
Olympic Trials (1)	2008	8
Total	1978–2017	283

**Table 2:** Descriptive statistics of demographic, anthropometric, and basic sports achievements in the entire group of athletes (*n* = 283).

Variables	x	SD	Min–Max	Median	Skewness	Kurtosis	*r*
Age (years)	26.05	3.38	18–37	26.05	0.54	0.45	−0.7 **
Body height (cm)	172.57	5.09	157–185	172	−0.30	0.62	−0.19 ***
Body mass (kg)	59.74	4.33	45–69	59	−0.08	0.15	−0.03
BMI	20.06	1.10	16.46–22.72	20.07	−0.19	0.07	−0.16 **
400 m H time (s)	54.53	0.86	52.16–56.90	54.6	0.56	0.33	-
PB 400 m (s)	51.93	1.22	49.24–56.91	51.90	0.36	0.43	0.36 ***
Time Index (s)	2.60	1.0	−1.71–5.20	2.62	−0.17	0.7	0.10

Time Index, the difference between the 400 m H time and the time of 400 m flat; ** *p* ≤ 0.01 for *r* = 0.16, *** *p* ≤ 0.001 for *r* = 0.19.

**Table 3:** Descriptive statistics of basic temporal variables (IHU) in the entire group of athletes.

Variable	x	SD	Min–Max	Median	Skewness	Kurtosis	*r*
*t*_0–1_	6.49	0.16	6.00–7.01	6.50	0.11	0.31	0.34
*t*_1–2_	4.18	0.13	3.86–4.60	4.20	0.22	−0.07	0.48
*t*_2–3_	4.27	0.13	4.00–4.70	4.26	0.48	−0.07	0.57
*t*_3–4_	4.36	0.13	4.00–4.86	4.35	0.49	0.72	0.55
*t*_4–5_	4.48	0.13	4.20–4.88	4.48	0.54	0.05	0.65
*t*_5–6_	4.62	0.14	4.40–5.10	4.60	0.65	0.07	**0.73**
*t*_6–7_	4.75	0.16	4.40–5.40	4.74	0.42	0.61	0.71
*t*_7–8_	4.94	0.18	4.50–5.60	4.95	0.48	0.94	0.71
*t*_8–9_	5.11	0.16	4.70–5.80	5.10	0.49	0.61	**0.75**
*t*_9–10_	5.25	0.18	4.80–6.00	5.27	0.38	0.54	0.68
*t*_10*–F*_	6.08	0.35	5.21–8.10	6.08	1.23	4.82	0.50

*t*, the time between particular hurdles; *F*, final time in 400 mH; *p* ≤ 0.05; significance is marked in bold.

**Table 4:** Descriptive statistics of basic and addition spatial variables (IHU) in the entire group of athletes.

Variable	x	SD	Min–Max	Median	Skewness	Kurtosis	*r*
*n*_1–2_	15.09	0.57	13–17	15	1.18	4.42	0.21 *
*n*_2–3_	**15.08**	0.56	14–17	15	1.35	4.15	0.18 *
*n*_3–4_	15.11	0.56	14–17	15	1.27	3.61	0.26 *
*n*_4–5_	15.15	0.57	14–17	15	1.23	2.92	0.30 *
*n*_5–6_	15.38	0.65	14–17	15	1.30	0.74	0.36 *
*n*_6–7_	15.60	0.71	14–17	15	0.71	–0.64	0.37 *
*n*_7–8_	16.00	0.78	14–18	16	0.10	–0.86	0.32 *
*n*_8–9_	16.38	0.75	15–18	16	0.20	–0.22	0.32 *
*n*_9–10_	16.65	0.74	15–19	17	0.01	0.31	0.29 *

*n*, means numbers of steps between particular hurdles; *p* ≤ 0.05, significance is marked in bold and *.

**Table 5:** Descriptive statistics of hurdle variable addition and differences (temporal and space).

Variable	x	SD	Min–Max	Median	Skewness	Kurtosis	*r*
*t*_1–4_	12.81	0.35	12.10–14.00	12.80	0.70	0.60	0.63 ***
*t*_4–7_	1.85	0.37	13.10–15.00	13.81	0.46	−0.14	0.82 ***
*t*_7–10_	15.30	0.46	14.40–17.40	15.29	0.52	0.87	0.81 ***
*n*_1–4_	45.28	1.63	41–51	45	1.34	4.17	0.22 **
*n*_4–7_	46.13	1.76	42–51	45	1.21	0.91	0.38 ***
*n*_7–10_	49.02	2.03	44–55	49	0.14	−0.23	0.34 ***
*t*_4–7_–*t*_1–4_	1.04	0.31	0.20–1.80	1.00	−0.03	−0.10	0.26 ***
*t*_7–10_–*t*_4–7_	1.45	0.39	0.60–2.90	1.44	0.32	0.49	0.19 **
*t*_7–10_–*t*_1–4_	2.50	0.51	0.90–3.90	2.50	0.32	−0.03	0.30 ***
*n*_4–7_–*n*_1–4_	0.84	1.03	0–4	0	0.84	−0.45	0.31 ***
*n*_7–10_–*n*_1–4_	3.74	1.69	0–10	4	0.37	0.45	0.19 **
*n*_7–10_–*n*_4–7_	2.90	1.41	0–7	3	0.22	0.10	0.01

*** *p* < 0.001; ** *p* < 0.01; significance is marked in bold.

**Table 6:** Simple r-Pearson’s correlation coefficients between all variables.

Variable	(1)	(2)	(3)	(4)	(5)	(6)	(7)	(8)
*t*_0–1_ (1)	-							
*t*_1–4_ (2)	0.45	-						
*t*_4–7_ (3)	0.19	0.60	-					
*t*_7–10_ (4)	0.01	0.23	0.54	-				
*t*_10–*F*_ (5)	0.30	0.16	0.07	0.51	-			
*n*_1–4_ (6)	0.20	0.17	0.12	0.08	0.02	-		
*n*_4–7_ (7)	0.11	0.22	**0.39**	0.32	0.15	0.79	-	
*n*_7–10_ (8)	0.05	0.07	0.17	**0.40**	0.31	0.59	0.76	-

Significance is marked in bold; *t*, time between particular hurdles; *n*, number of steps between particular hurdles.

**Table 7:** Stepwise forward regression coefficients. Parameter of regression model for the dependent variable (T400 mH).

Variables	BETA	Standard Error	Standard BETA	*t*	*p*
Intercept	−1.015	1.065	0.338	−0.953	0.342
*t*_4–7_	**0.93**	0.069	0.410	14.463	**0.000**
*t*_7–10_	**0.975**	0.051	0.243	19.220	**0.000**
*t*_1–4_	**0.783**	0.066	0.316	11.924	**0.000**
*t*_10–*F*_	**0.987**	0.057	0.188	17.413	**0.000**
*t*_0–1_	**1.204**	0.112	0.078	10.774	**0.000**
Time Index	0.076	0.031	0.019	2.470	**0.014**
*n*_1–4_	0.013	0.010	0.036	1.284	0.201
*n*_4–7_	0.010	0.004	−0.025	2.404	**0.017**
*n*_7–10_–*n*_1–4_	−0.015	0.010	0.338	−1.526	0.129
PB400 m	0.040	0.029	0.047	1.358	0.176

Parameter of regression model: *R* = 0.981; *R*^2^ = 0.962; corrected *R*^2^ = 0.960; *p* ≤ 0.05; significance is marked in bold; *t*, time between particular hurdles; *n*, numbers of steps between particular hurdles.

**Table 8:** PCA analysis of selected 400 m hurdles strategy variables.

Variable/Factor	1	2	3	4	5	6	Importance of the Variables	
Age	0.07	0.04	0.33	−0.40	0.34	0.19	0.42	20
Body Height	0.26	−0.28	−0.36	−0.28	**0.50**	−0.35	0.72	15
Body Mass	0.15	**−0.55**	−0.20	−0.23	**0.56**	0.04	0.73	13
BMI	−0.10	−0.41	0.14	−0.01	0.16	0.46	0.43	19
Personal Best 400 m	−0.27	0.38	−0.17	**−0.77**	−0.32	0.16	0.94	**2**
Time Index	0.15	0.06	−0.13	**0.79**	0.40	−0.33	0.92	**3**
*t*_0–1_	0.20	**0.55**	0.02	−0.15	0.12	−0.44	0.57	17
*t*_1–4_	0.14	**0.73**	−0.17	−0.29	0.20	−0.28	0.79	11
*t*_4–7_	−0.32	**0.56**	**−0.69**	−0.07	0.09	0.03	0.90	**5**
*t*_7–10_	**−0.80**	0.12	−0.30	−0.08	−0.12	−0.36	0.90	**5**
*t*_10*–F*_	**−0.67**	−0.18	0.02	−0.03	−0.07	−0.09	0.50	18
*t*_4–7_–*t*_1–4_	**−0.51**	−0.14	**−0.60**	0.23	−0.12	0.33	0.82	10
*t*_7–10_–*t*_4–7_	**−0.63**	−0.36	0.27	−0.02	−0.22	−0.46	0.86	8
*t*_7–10_–*t*_1–4_	**−0.81**	−0.37	−0.16	0.12	−0.24	−0.15	0,.1	**4**
*n*_1–4_	−0.05	**0.70**	0.42	0.21	−0.07	0.05	0.72	14
*n*_4–7_	−0.38	**0.75**	0.17	0.24	0.16	0.23	0.87	7
*n*_7–10_	**−0.70**	0.43	0.46	0.06	0.28	0.08	0.96	**1**
*n*_4–7_–*n*_1–4_	**−0.56**	0.18	−0.36	0.09	0.38	0.30	0.72	16
*n*_7–10_–*n*_1–4_	**−0.80**	−0.12	0.16	−0.11	0.41	0.05	0.82	9
*n*_7–10_–*n*_4–7_	**−0.56**	−0.30	0.48	−0.21	0.22	−0.18	0.74	12
Own value %	4.67	3.56	2.20	1.85	1.63	1.45		
	23.36	17.79	11.00	9.27	8.15	7.25		
Cumulative eigenvalue %	4.67	8.23	10.43	12.28	13.91	15.36		
	23.36	41.15	52.15	61.42	69.57	76.82		

1. Factor, the third part of the distance; 2. Factor, the first part of the distance; 3. Factor, the second part of the distance; 4. Factor, Motor and technical factor; 5. Factor, Body build factor; 6. Factor, Nonqualified factor. Significance is marked in bold; *t*, time between particular hurdles; *n*, number of steps between particular hurdles.

**Table 9:** Mean and SD values of variables in sports level categories and results of ANOVA analysis with *F* and *p*-values. Post hoc statistical significance for detailed comparisons.

Variables	Groups of Hurdles			*F*	*p*	Post Hoc		
	Group A	Group B	Group C			A–B	A–C	B–C
Time 400 mH	53.38 (0.42)	54.51 (0.29)	55.83 (0.67)	612.0	0.001	xxx	xxx	xxx
Age	26.94 (3.68)	25.63 (3.06)	25.14 (3.70)	5.12	0.05		xx	
Body Height	172.56 (5.23)	172.76 (5.36)	172.26 (5.52)	0.23	NS			
Body Mass	60.60 (4.34)	59.74 (4.10)	58.93 (4.48)	3.49	0.05		xx	
BMI	20.34 (1.00)	20.02 (1.11)	19.86 (1.16)	4.70	0.01	x	xx	
Personal Best 400 m	51.34 (1.14)	51.99 (1.08)	52.44 (1.24)	20.31	0.001	xxx	xxx	xx
Time Index	2.04 (1.37)	2.52 (1.66)	3.39 (1.46)	9.93	0.001	xx	xxx	xx
*t*_0–1_	6.45 (0.15)	6.48 (0.16)	6.56 (0.16)	9.50	0.001		xxx	xx
*t*_1–4_	12.58 (0.22)	12.76 (0.23)	13.13 (0.35)	82.15	0.001	xxx	xxx	xxx
*t*_4–7_	13.52 (0.20)	13.83 (0.22)	14.23 (0.28)	177.40	0.001	xxx	xxx	xxx
*t*_7–10_	14.89 (0.28)	15.37 (0.30)	15.69 (0.36)	127.39	0.001	xxx	xxx	xxx
*t*_10*–F*_	4.98 (0.11)	5.11 (0.13)	5.24 (0.58)	21.67	0.001	xxx	xxx	xxx
*t*_4–7_–*t*_1–4_	0.94 (0.25)	1.07 (0.30)	1.11 (0.36)	6.36	0.001	xx	xxx	
*t*_7–10_–*t*_4–7_	1.37 (0.33)	1.51 (0.39)	1.46 (0.44)	3.01	NS			
*t*_7–10_–*t*_1–4_	2.31 (0.42)	2.58 (0.47)	2.57 (0.58)	7.86	0.001	xxx	xxx	
*n*_1–4_	45.02 (.25)	45.26 (1.23)	45.63 (1.84)	2.72	0.05		x	
*n*_4–7_	45.56 (1.23)	46.10 (1.73)	46.90 (2.05)	12.40	0.001	x	xxx	xx
*n*_7–10_	48.39 (1.63)	49.12 (1.99)	49.72 (2.32)	8.95	0.001	x	xxx	
*n*_4–7_–*n*_1–4_	0.54 (0.82)	0.84 (0.98)	1.27 (1.17)	10.77	0.001	x	xxx	xx
*n*_7–10_–*n*_1–4_	3.37 (1.40)	3.86 (1.68)	4.09 (1.94)	3.85	0.05	x	xxx	
*n*_7–10_–*n*_4–7_	2.83 (1.22)	3.02 (1.51)	2.82 (1.49)	0.60	NS			
*n*	80	94	98	Total	283 runs			

*t*, the time between particular hurdles; *n*, numbers of steps between particular hurdles, x, *p* < 0.05; xx, *p* < 0.01; xxx, *p* < 0.001; NS, no relationship.

## Data Availability

The data presented in this study are openly available on: https://wwfif.po.edu.pl/ (accessed on 16 January 2022) and on request from the first author.

## References

[1] Schache A.G., Wrigley T.V., Baker R., Pandy M.G. (2009). Biomechanical response to hamstring muscle strain injury. Gait Posture.

[2] Carpes F.P., Mota C.B., Faria I.E. (2010). On the bilateral asymmetry during running and cycling—A review considering leg preference. Phys. Ther. Sport.

[3] Ciacci S., Di Michele R., Fantozzi S., Merni F., Mokha M. (2013). Assessment of kinematic asymmetry for reduction of hamstring injury risk. Int. J. Athl. Ther. Train..

[4] Bishop C., Read P., Lake J., Chavda S., Turner A. (2018). Inter-limb asymmetries: Understanding how to calculate differences from bilateral and unilateral tests. Strength Cond. J..

[5] Madruga-Parera M., Bishop C., Read P., Lake J., Brazier J., Romero-Rodriguez D. (2020). Jumping-based asymmetries are negatively associated with jump, change of direction, and repeated sprint performance, but not linear speed, in adolescent handball athletes. J. Hum. Kinet..

[6] Zifchock R.A., Davis I., Hamill J. (2006). Kinetic asymmetry in female runners with and without retrospective tibial stress fractures. J. Biomech..

[7] Laroche D.P., Cook S.B., Mackala K. (2012). Strength asymmetry increases gait asymmetry and variability in older women. Med. Sci. Sports Exerc..

[8] Vagenas G., Hoshizaki B. (1991). Functional asymmetries and lateral dominance in the lower limbs of distance runners. Int. J. Sport Biomech..

[9] Lundberg Zachrisson A., Ivarsson A., Desai P., Karlsson J., Grau S. (2020). Athlete availability and incidence of overuse injuries over an athletics season in a cohort of elite Swedish athletics athletes—A prospective study. Inj. Epidemiol..

[10] Jacobsson J., Timpka T., Kowalski J., Nilsson S., Ekberg J., Renström P. (2012). Prevalence of musculoskeletal injuries in Swedish elite track and field athletes. Am. J. Sports Med..

[11] Quercetani R.L. (2009). A World History of Hurdle and Steeplechase Racing.

[12] Gupta S., Goswami A., Mukhopodhayay S. (1999). Heart rate and blond lactate in 400 m flat and 400 m hurdles running. A comparative study. Indian J. Physiol. Pharmacol..

[13] Zauhal H., Jabbour G., Jacob C., Duvigneau D., Botcazou M., Abderrahman A., Prioux J., Moussa E. (2010). Anaerobic and aerobic energy system contribution to 400-m flat and 400-m hurdles track running. J. Strength Cond. Res..

[14] Guex K. (2012). Kinematic analysis of the women’s 400 m hurdles. New Stud. Athl..

[15] Reis V.M., Duarte J.A., Espírito-Santo J., Russell A.P. (2004). Determination of accumulated oxygen deficit during a 400 m run. J. Exerc. Physiol. Online.

[16] McFarlane B. (2004). The Science of Hurdling and Speed.

[17] Iskra J. (2012). Athlete typology and training strategy in the 400 m hurdles. New Stud. Athl..

[18] Hiserman J.A. (2008). Program Design Method for Sprint & Hurdle Training.

[19] Iskra J. (2013). Advanced Training in the Hurdles.

[20] Behm J.J. (2016). Quatrache 400 Haies Histoire du Monde 1900–2016 (Quatrache 400 Hurdles World History 1900–2016).

[21] Ditroilo M., Marini M. (2000). Analysis of the race distribution for male 400 m hurdles competing at the 2000 Sydney Olympic Games. New Stud. Athl..

[22] Hiserman J. (2011). The Art of Long Hurdling. Obtained from Speed Endurance. http://speedendurance.com/.

[23] Iskra J. (2008). Changes of stride pattern of world class 400-m hurdlers–reasons and consequences. International Convention on Science, Education and Medicine in Sport, Guangzhou, China, 1–5 August 2008.

[24] Iskra J. (2012). Badania naukowe w biegach przez płotki. Scientific Research in Hurdles.

[25] Iskra J., Walaszczyk A. (2007). Types of stride pattern and time distribution in elite 400-m hurdlers. Motor Control..

[26] Iskra J., Čoh M. (2011). Biomechanical studies on running the 400 m hurdles. Hum. Mov..

[27] Pietrzak M., Iskra J. (2016). Changes in women’s 400 m hurdle run from 1978 to 2014. Phys. Act. Rev..

[28] Bankin W.N. (1995). Analysis of the competitive activity of young runners of various qualifications in the 400 m hurdles. Teoria I Praktika Fiziczeskoj Kultury.

[29] Greene D., Leyshon W., O’Donoghue P.G. (2008). Elite male 400 m hurdle tactics are influenced by race leader. World Congress of Performance Analysis of Sport.

[30] Iskra J., Cilik I., Pupis M. (2019). In search of temporal characteristic in men’s 400 m hurdle run. Atletika 2019.

[31] Yasui T., Ogiso K., Aoyama K., Sekioka Y., Nagai J., Miyashita K., Ogata M. (1997). The analysis of race in 400 M hurdle for woman. 14th International Society Biomechanics Congress, Book of Abstracts.

[32] Gangxu L.U., Xigen H. (2007). Velocity characteristics of world elite women’s 400 m hurdles. J. Shandong Inst. Phys. Educ. Sport.

[33] Qiuhua H.E., Xinfa T., Ciaohong Y. (2002). The space-temporal features of pacing rhythm of Chinese super women 400-hurdle racers. Sichuan Sports Sci..

[34] Beckenham M., Rosemond D. (2006). Establishing an ideal stride pattern for a potential elite female 400 m hurdle. Mod. Athl. Coach.

[35] Letzelter H., Letzelter M. (1979). Zum Einfluss von Kondition und Technik beim 400-m-Hürdenlauf der Männer und Fauen. (On the influence of fitness and technique in the men’s and women’s 400 m hurdles). Leistungssport.

[36] Xinyong W., Manyun Z. (2003). Analysis on the rhythm of stride in male 400 m hurdles. J. Anhui Sports Sci..

[37] Matousek R., Sedlacek J. (1987). Relation between the performance in the men’s 400 m hurdles and the running speed between the hurdles. Teor. Praxe Teles. Vych..

[38] Letzelter H. Stride pattern in the 400 m hurdles for women. Proceedings of the Women’s Track and Field Athletics. 1st IAAF Congress on Women’s Athletics.

[39] Iskra J., Matusiński A., Otsuka M., Guex K. (2021). Pacing strategy in men’s 400 m hurdles accounting for temporal and spatial characteristics of elite athletes. J. Hum. Kinet..

[40] Lopez V. (1998). Practical analysis of the results of the 400 metres hurdles for women. IAAF Salinas Bull..

[41] Federolf P., Reid R., Gilgien M., Hauger P., Smith G. (2012). The application of principal component analysis to quantify technique in sports. Scand. J. Med. Sci. Sports.

[42] Iskra J. (2003). Próba zastosowania analizy czynnikowej do oceny parametrów kinemetycznych kroku płotkowego (An attempt to use factor analysis to evaluate the kinetic parameters of the fence step). Antropomotoryka.

[43] Rovniy A., Pasko V., Stepanenko D., Grebieniuk O. (2017). Hypoxic capacity as the basis for sport efficiency achievements in the men’s 400-meter hurdling. J. Phys. Educ. Sport.

[44] Ozaki Y., Ueda T., Fukuda T., Inai T., Kido E., Narisako D. (2019). Regulation of Stride Length During the Approach Run in the 400-M Hurdles. J. Hum. Kinet..

